# Association between catastrophic health expenditure and mortality in older people in 11 European health systems: a longitudinal analysis between 2006 and 2020

**DOI:** 10.1136/bmjph-2025-003228

**Published:** 2025-11-13

**Authors:** Raffaele Palladino, Christopher Millett, Michele Sorrentino, Michelangelo Mercogliano, Paolo Montuori, Thomas Hone

**Affiliations:** 1 Department of Primary Care and Public Health, Imperial College London, London, UK; 2 Department of Public Health, University “Federico II” of Naples, Naples, Italy; 3 Interdepartmental Research Center on Management and Innovation in Healthcare, University “Federico II” of Naples, Naples, Italy; 4 NOVA National School of Public Health, Public Health Research Centre, Comprehensive Health Research Center, NOVA University Lisbon, Lisbon, Portugal

**Keywords:** Epidemiology, economics, Health Services Accessibility, Public Health

## Abstract

**Introduction:**

Out-of-pocket expenditure (OOPE) and catastrophic health expenditure (CHE) challenge the financial protection provided by European health systems. Despite rising OOPE and CHE in Europe, evidence on health impact remains limited. This study examines the association between OOPE, CHE and all-cause mortality in older adults across 11 European countries from 2006 to 2020.

**Methods:**

We analysed longitudinal data from the Survey of Health, Ageing and Retirement in Europe, including 70 367 individuals aged ≥50 years across five waves (wave 2 (2006/2007), 5 (2013), 6 (2015), 7 (2017) and 8 (2019/2020)). CHE was defined as annual OOPE exceeding 25% of household income. Parametric survival models with Gompertz distribution estimated the association between CHE, OOPE and mortality, adjusting for socioeconomic and clinical covariates. Sensitivity analyses employed inverse probability weighting with regression adjustment.

**Results:**

Participants had a mean age of 65 years, 54% were women and 46% were retired. CHE was incurred by 1.6% of participants (range: 0.9%–2.2% by country). Over a mean follow-up of 3.8 years, 10.2% (7193) died. CHE was associated with an 84% increased mortality risk (95% CI 1.66 to 2.04), with the strongest effect in Switzerland (HR 4.22, 95% CI 2.54 to 7.00). Each €100 increase in total OOPE was associated with a 0.4% higher mortality risk (95% CI 1.002 to 1.006), especially in higher-income groups. OOPE on medicines showed heterogeneous effects: in the lowest income quintile, higher spending was associated with reduced mortality (HR 0.955, 95% CI 0.927 to 0.982), while in the third quintile (HR 1.044, 95% CI 1.017 to 1.071) and in countries like Czechia (HR 1.086, 95% CI 1.017 to 1.159) and Germany (HR 1.015, 95% CI 1.003 to 1.028), it was associated with increased mortality. Sensitivity analyses confirmed the main results.

**Conclusions:**

CHE and elevated OOPE are independently associated with increased mortality in older Europeans, suggesting negative long-term impacts on health. These findings underscore the need for cost-sharing reforms to preserve equity and protect vulnerable populations.

**WHAT IS ALREADY KNOWN ON THIS TOPIC**

**WHAT THIS STUDY ADDS**

- This study demonstrates a robust longitudinal association between CHE and increased mortality across 11 European countries (greater risk found in Switzerland). An increase in OOPE exhibited similar but weaker positive associations. The effect of OOPE on medicines varies by income level.

**HOW THIS STUDY MIGHT AFFECT RESEARCH, PRACTICE OR POLICY**

- Findings highlight the need to reassess cost-sharing policies and monitor financial protection indicators. Policymakers should consider equity impacts when designing mechanisms to finance healthcare, especially for older adults.

## Introduction

 Out-of-pocket expenditures (OOPE) on healthcare are a major impediment to universal health coverage (UHC). The OOPE is increasing worldwide and in western countries like the USA,^1^ but it remains a challenge even in European countries, despite widespread coverage under universal systems with substantial government financing. In 2021, 14.5% (€245 billion) of the €1592 billion total expenditure on health in the European Union (EU) was OOPE.^2^ Households may encounter financial strain when OOPE is high. Catastrophic health expenditure (CHE) represents the most severe form of OOPE—commonly defined as exceeding a benchmark of household income (often 10%, 25% or 40%).^3 4^ While CHE continues to remain an issue in the USA and other western countries,^5^ in the EU countries, on average, 4% of households incurred catastrophic CHE in 2021—a proportion which increased in recent years.^6^

OOPE primarily stems from outpatient medicines, medicinal products and dental care,^6^ with medicine expenditures being the largest global source, especially among older individuals with multimorbidity.^7^,^9^ Low-income populations are disproportionately affected and more likely to incur CHE.^6^ The interplay between health status, healthcare use and OOPE is complex. Since OOPE is tied to healthcare utilisation, those with chronic or costly conditions pay more.^810^,^12^ High costs for healthcare can also result in forgone healthcare,^13^ masking the true impacts of poor financial risk protection.

Increasing OOPE, and the resulting risk of incurring CHE, can push households into poverty. This reduces funds for other health-improving activities (eg, housing, sanitation, nutrition, exercise), prohibiting purchasing further costly healthcare or medicines. If health issues remain unresolved, households may also face lost income due to an inability to work. This can negatively affect individuals’ health in the longer term. Despite evidence demonstrating the negative impacts of OOPE and CHE—such as household impoverishment,^14^ poor quality of life^15^ and mental health^16^—few studies have assessed the longer-term risks of poor health outcomes after incurring CHE. This analysis is complex because both underlying health status and the ability to pay for healthcare influence OOPE and confound assessments of long-term health risks.

European health systems are frequently lauded for their progress towards UHC and low levels of OOPE and CHE.^17^ However, European health systems are facing sharp budgetary crises from declining macroeconomic conditions, ageing populations and rising healthcare costs.^6^ Notably, the high and escalating costs of medicines—often misaligned with demonstrable health gains—have intensified fiscal pressures.^18^ In response, many countries in Europe have introduced cost-sharing policies in recent years to rationalise demand and raise income—particularly for medicines.^7 8^

This study assesses the relationship between CHE and mortality in older people in 11 European countries using a longitudinal data analysis between 2006 and 2020. It also assesses the relationship between OOPE (the main driver of CHE), OOPE on medicine, specifically, and mortality. The longitudinal design allowed us to follow the same individuals over multiple years, accounting for changes in exposure and covariates, to assess their risk of death.

## Methods

### Study design

This study is a longitudinal data analysis. It analyses individuals aged 50 years and older from 11 European health systems whose information was recorded in at least two waves of the Survey of Health, Ageing and Retirement in Europe (SHARE) between 2006 and 2020.^19^

### Data and sample

We used data from wave 2 (2006/2007), 5 (2013), wave 6 (2015), wave 7 (2017) and wave 8 (2019/2020) of SHARE, a European panel database containing nationally representative samples of respondents aged 50 and over from 28 European countries and Israel. Respondents’ sociodemographic factors, health status (including the presence of chronic illnesses and disability) and healthcare use and spending are all included in the data. SHARE’s methodologies have been described in depth elsewhere.^20 21^ Data from the following 11 countries were included because they participated in all waves included in the present analysis: Austria, Belgium, Czechia, Denmark, France, Germany, Italy, Netherlands, Spain, Sweden and Switzerland. Considering the comparable levels of service coverage among the selected European health systems—as indicated by UHC Service Coverage Index scores exceeding 80 for all countries in 2021^22^—analyses were performed both collectively (by pooling all countries) and individually for each country. We included data on all individuals whose information was recorded in at least two waves. Data from waves 3 and 4 were not included in this analysis, as no information on OOPE was collected in these waves.^23 24^ Although wave 8 completion was disrupted by the COVID-19 pandemic, from this specific database, we only retrieved information on the death of participants from previous waves. We generated an unbalanced sample of 70 367 individuals with 159 832 observations from the waves.

### Variables

#### Outcome

The study outcome was all-cause mortality. Participants’ dates of death were obtained from waves 5 to 8 and used in the analysis. In line with previous studies,^25^ date of death was obtained through an end-of-life interview with a proxy respondent for all participating households.^2324 26^,^29^ Survival time was calculated as the duration between the initial interview date and either the date of death for deceased participants or the final interview date for those not deceased.

#### Variables of interest

CHE was the main variable of interest. We used data on household healthcare expenditures and income to calculate CHE. To calculate the household income, information on employment income, pension income, social benefits and capital income was considered.^2324 26^,^29^ In line with the World Bank definition,^4^ we defined CHE as OOPE for healthcare (for services and medicines) exceeding 25% of the household income. This threshold was selected as it is internationally recognised,^4^ aligns with Sustainable Development Goal indicator 3.8.2 and provides a comparable measure of financial burden.

We calculated OOPE to construct our CHE variable but also included OOPE (total and for medicines) as secondary variables of interest. Overall OOPE was calculated as the sum of OOPEs on medicines, outpatient and inpatient services. We also specifically examined OOPE on medicines as the majority of the cost-sharing policies introduced in European health systems focus on medicines,^7 8^ and OOPE on medicine accounts for the largest source of OOPE globally—especially in older people with multimorbidity.^7^,^9^ An example of the questions used to construct this variable was: ‘About how much did you pay altogether for drugs in the last twelve months? (Include both doctor-prescribed and non-prescription drugs)’. OOPE was expressed in € and was adjusted for country-specific inflation to the year of the latest data collection (2020) to allow comparisons across time.

#### Covariates

To adjust the relationship between CHE and mortality for potential confounding from underlying health status and socioeconomic factors, we included the following covariates: age (50–59, 60–69 or 70 and older); sex (male, female); body mass index (BMI) (continuous); marital status (married or in a civil partnership, others); country of residence; educational attainment (less than upper secondary, upper secondary or tertiary education); household income quintile (the poorest being Q1, the richest being Q5); the number of primary care visits and hospitalisations in the previous year (counts) and multimorbidity expressed as the number of coexisting chronic diseases (CDs) reported by each respondent. To assess multimorbidity, we considered 15 CDs, 14 self-reported health conditions (heart attack/problem, hypertension, hypercholesterolaemia, stroke/cerebral vascular illness, diabetes, cancer, peptic, stomach or duodenal ulcer, chronic lung disease, arthritis/rheumatism or osteoarthritis, Parkinson’s disease, cataracts, hip or femoral fracture, other fracture, Alzheimer disease/dementia/organic brain syndrome/senility/other significant cognitive impairment and one symptom-based health condition (depression). Participants were asked to answer the following question: ‘Has a doctor ever told you that you had/do you currently have any of the conditions listed on this card?’. Clinical depression was the only chronic disease that was not defined based on the answer to this question. The EURO-D scale was used to measure and define it, in agreement with prior studies,^11 30^ with scores of 4 or higher indicating the presence of clinically significant depressive symptoms.

### Statistical analyses

To avoid loss of significant amount of information, we used the imputed data provided by the SHARE team. Details and methods have been published elsewhere.^2426^,^28 31^ Briefly, for variables affected by negligible fractions of missing values (much less than 5%), the hot-deck method was employed (ie, randomly replacing missing values with the observed values in the same variable from a respondent who has same values in auxiliary variables selected for both donor and recipient). For variables with larger proportions of missing data, the fully conditional specification method (FCS) of van Buuren *et al* was employed to deal with the missing data.^32 33^ In the present analysis, the variable with missing data that required the FCS method for imputation was household income (46.8% of missing data).

Population characteristics were described at baseline using proportion and means (with SD), as appropriate, and applying sample weights provided by SHARE. The study baseline was defined for each respondent as the first year of data availability. For each country, we reported the number of participants, and information on respondents’ socioeconomic and health characteristics, and healthcare utilisation. We graphically reported the prevalence of CHE and mean OOPE (total and on medicines) for each of the 11 countries for the study period.

We modelled the association between CHE and mortality employing parametric survival models with Gompertz distribution. The Gompertz specification implies an exponentially increasing hazard with age, which is concordant with adult human mortality and is widely used in demography and gerontology.^2534^,^36^ In older populations, this assumption often provides a close approximation to the underlying mortality process, enabling more efficient estimation and straightforward absolute risk predictions compared with semi-parametric approaches.^2537^,^39^ The choice of Gompertz over Cox reflected the plausibility of a monotone, accelerating baseline hazard in our cohort. Parametric survival models can be more efficient when the baseline hazard has a credible functional form, whereas Cox leaves the baseline unspecified.^39^ The Gompertz model was also preferred to the Cox proportional hazards model based on statistical assessments using the Akaike Information Criterion and Cox-Snell residuals.^25^ Statistical analyses were controlled for the covariates listed above. Time-varying covariates included household BMI, household income quintile, number of primary care visits and hospitalisations in the previous year and number of CDs. These were updated across survey waves for each individual, allowing the model to reflect changes in covariate values over time that might influence the association with the study outcome. This was implemented through episode splitting, enabling dynamic estimation of risk over the follow-up period. Analyses were carried out for a pooled sample of all countries (additionally adjusting for country fixed effects with a dummy variable for each country) and then in separate models for each income quintile and country. These stratified analyses allowed us to estimate whether there was variation in the relationship between CHE and mortality across countries and income groups. To account for the longitudinal structure of the data, SEs were clustered at the individual level to control for serial correlation.

Finally, we assessed the relationship between total OOPE and OOPE on medicine and mortality using the same models. We reported results for a pooled analysis of all countries and stratified models by income quintiles and country. Results were presented as HRs and 95% CIs. For models including OOPE, coefficients were reported per each €100 increase in OOPE expenditure.

Analyses were performed using Stata MP, V.17.0.

### Sensitivity analyses

As the likelihood of incurring CHE is generally higher in individuals from lower socioeconomic groups and of poorer health status, there may be selection bias affecting the association between CHE and mortality. To assess the robustness of the main analysis, we repeated our analyses for CHE and mortality including the inverse probability weighting regression adjustment (IPW-RA) method. The IPW-RA model accounted for the probability in incurring CHE considering the sociodemographic and clinical characteristics of respondents. The propensity score of encountering CHE was calculated using logit models adjusted for age, gender, marital status, education attainment, household income, number of chronic diseases, number of primary care visits and hospitalisations in the previous year, and country of residence. Although doubly robust methods are widely used in observational studies,^40^ we considered the IPW-RA model a sensitivity analysis because IPW- RA can trade bias for variance. In settings with relatively infrequent exposure (CHE), limited covariate overlap (where treatment and control groups share few similar covariate patterns, approaching a violation of the assumption that everyone has some chance of receiving either treatment), or complex propensity models, the inverse probability weights can become highly variable, inflating SEs and producing wider CIs.^40^,^43^ In our data, this pattern likely reflects variance introduced by weighting rather than a qualitative change in the association. We therefore present IPW- RA as a sensitivity analysis, and we report the main parametric survival estimates—supported by modelfit diagnostics—as our primary results.

### Patient and public involvement

As this study is a quantitative secondary data analysis using large-scale survey data (eg, SHARE), patient and public involvement (PPI) was not included in the development or execution of this study. The research relied on pre-existing, anonymised data collected through standardised procedures, limiting the feasibility and relevance of PPI.

## Results

Data on 70 367 participants were included (table 1, online supplemental table 1). Mean follow-up time was 3.8 (SD 3.4 years; upper limit 13 years), with respondents from Denmark contributing the longest follow-up (4.7; SD 3.6 years) and those from the Netherlands the shortest (1.9; SD 2.8 years). At baseline, participants were, on average, 65 years old (mean age 65.0; SD 19.8), 54% were women, 67% were married and 46% were retired. The mean number of CDs at baseline was 1.6 (SD 2.6), and mean BMI was 26.5 (SD 7.7). The mean number of primary care visits and hospitalisation individuals reported in the previous 12 months was 7.5 (SD 17.9) and 0.3 (SD 1.3), respectively. The country with the highest primary care utilisation was Germany (8.6 consultations in last 12 months (SD 14.4), while the country with the lowest levels was Denmark (4.7; SD 7.8).

**Table 1:** Descriptive statistics of the study population at baseline

	Overall
n	70 367
Mean follow-up (years)	3.8 (3.4)
Age (mean (SD))	65.0 (19.8)
Gender (female)	53.7%
Marital status	
Married/partnership	67.0%
Single	7.9%
Divorced/widowed	25.2%
Education attainment	
No formal education	6.2%
Primary school	20.4%
Secondary and higher	73.4%
Income	
Q1 (poorest)	21.2%
Q2	18.9%
Q3	19.2%
Q4	19.7%
Q5 (richest)	21.0%
Job status	
Retired	46.3%
Employed/self-employed	32.1%
Unemployed	4.0%
Permanently sick/disabled	3.4%
Homemaker/other	14.2%
BMI (mean (SD))	26.5 (7.7)
Number of CDs (mean (SD))	1.6 (2.6)
Number of primary care visits in previous year (mean (SD))	7.5 (17.9)
Number of hospitalisations in previous year (mean (SD))	0.3 (1.3)

Notes: all individuals whose information was recorded in at least two waves starting from wave 2 (2006/2007) were included in the study. For each participant, the year of the first available interview was considered as baseline year. Cross-sectional survey weights were applied.

BMI, body mass index; CD, chronic disease.

**Figure 1:**
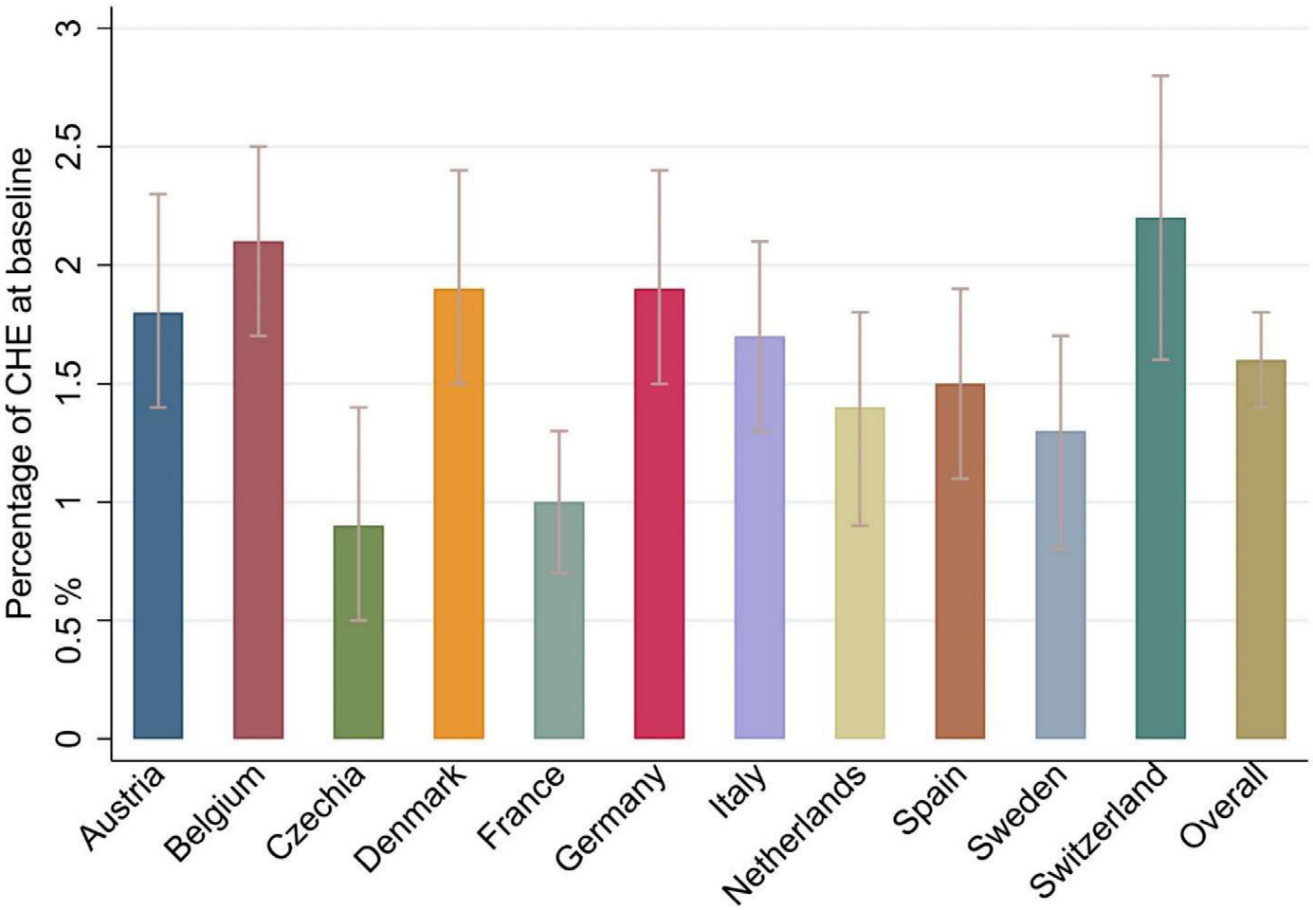
Catastrophic health expenditure across 11 European countries at baseline. Notes: catastrophic health expenditure was defined as expenditure for healthcare exceeding 25% of the household income. For each participant, the year of the first available interview was considered as baseline year. Cross-sectional survey weights were applied. CHE, catastrophic health expenditure.

**Figure 2:**
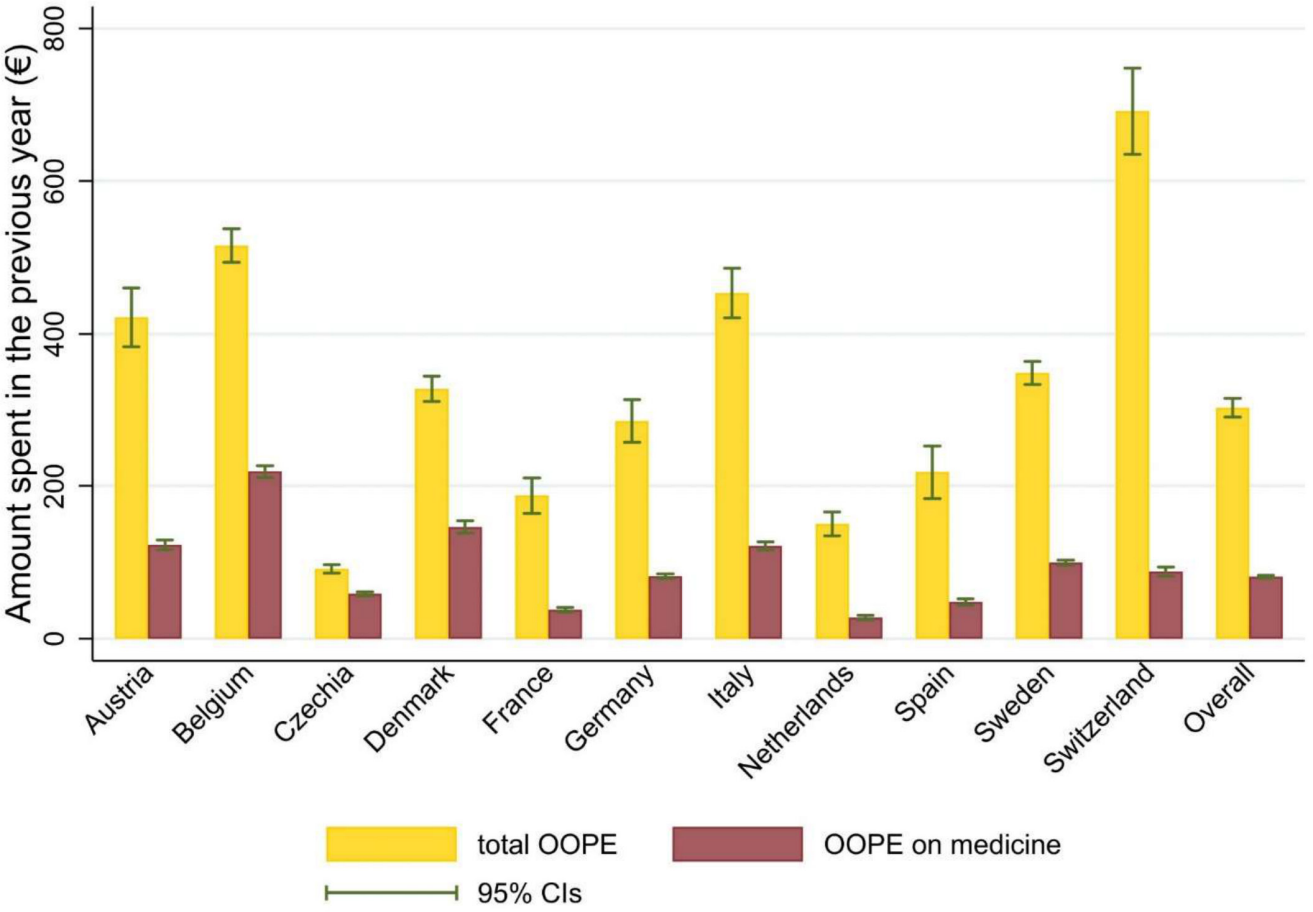
Out-of-pocket expenditure at baseline for the study population. Notes: out-of-pocket expenditure was calculated as the amount spent in the year before the baseline interview for each participant. Survey weights were applied. OOPE, out-of-pocket expenditure.

At baseline 1.6% (95% CI 1.4% to 1.8%) of participants incurred any CHE, with Switzerland being the country that reported the highest proportion (2.2%, 95% CI 1.6% to 2.8%) and Czechia reporting the lowest (0.9%, 95% CI 0.5% to 1.4%; figure 1). Respondents spent, on average, €303 (95% CI 291.2 to 315.6) in total OOPE and €81 (95% CI 79.6 to 82.9) in OOPE on medicine in the last 12 months. OOPE on medicines accounted for 26.7% of total OOPE. Respondents in Switzerland reported the largest amount of total OOPE (€691.6, 95% CI 635.2 to 748.0), while the lowest was reported in Czechia (€91.2, 95% CI 85.8 to 96.6). In Denmark and Belgium, nearly 45% of the total OOPE was spent on medicine. Full results are shown in figure 2 and in online supplemental table 2.

Over the study period, 10.2% (7193) of the participants died. The largest proportion of deaths (1274, 16.1%) was registered in Spain, while the lowest was in Austria (503, 9.9%).

**Figure 3:**
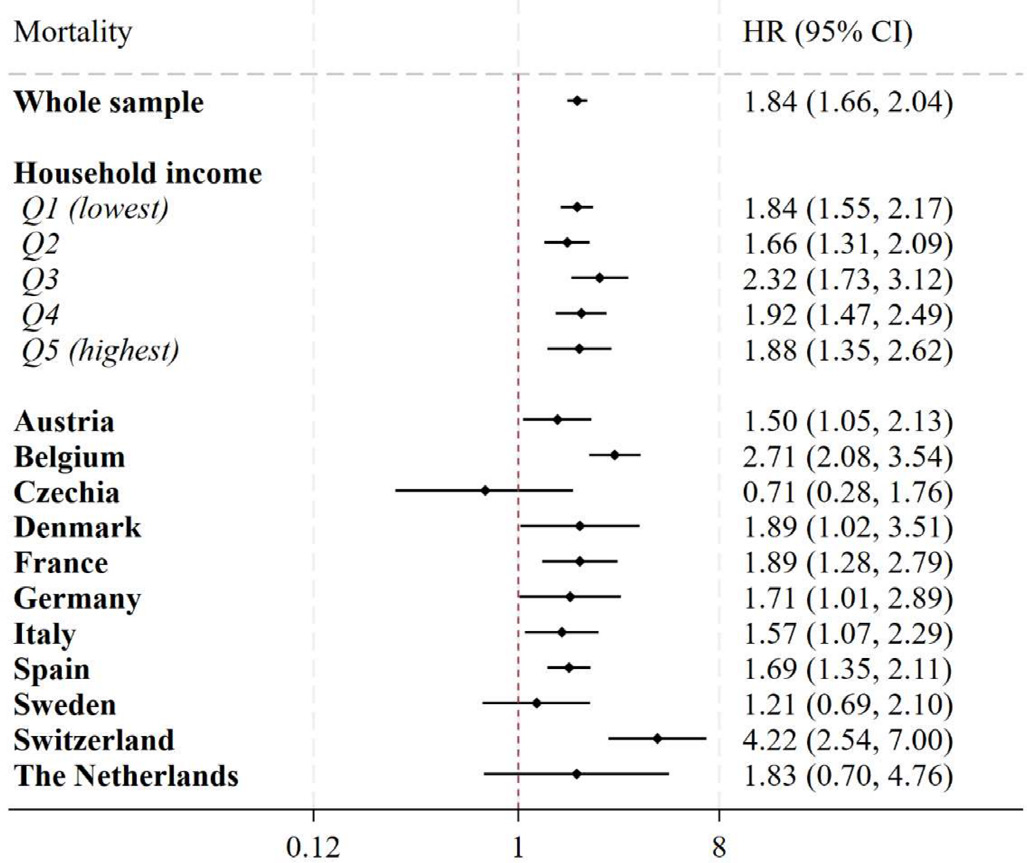
Association between catastrophic health expenditure and mortality in 11 European countries. Notes: the association between catastrophic health expenditure and mortality was modelled using time-varying parametric survival models with Gompertz distribution. Covariates included in the main models were: age (50–59, 60–69 or 70 and older), sex (male, female), BMI (continuous), number of chronic diseases (continuous), marital status (married or in a civil partnership, others), residential country, educational attainment (less than upper secondary, upper secondary or tertiary education), household income (in quintiles within each country for each wave, respectively; the poorest being Q1, the richest being Q5), number of primary care visits and hospitalisations in the previous year. Each point estimate and relative CIs in the graph were derived from a separate regression model. BMI, body mass index.

In adjusted parametric survival models including all countries (figure 3), incurring CHE in the previous year was associated with an 84% increase in the risk of mortality (HR 1.84, 95% CI 1.66 to 2.04). In stratified models by income quintile, CIs overlapped across quintiles. In models stratified by countries (figure 3), positive significant associations between CHE and mortality were found in all countries except for Czechia, Sweden and the Netherlands, where the associations were non-significant. Incurring CHE in Switzerland was associated with a 4.2-fold increase in the mortality risk (4.22, 95% CI 2.54 to 7.00), while the risk was 1.5 in Austria (95% CI 1.05 to 2.13).

In models assessing the relationship between total OOPE and mortality (pooled for all counties), each €100 increase in total OOPE was associated with a 0.4% increase in the risk of mortality over the study period (HR 1.004, 95% CI 1.002 to 1.006). In income quintile-stratified models, there was a clear trend of increasing mortality associated with increasing household income. There was no significant association between total OOPE and mortality in Q1, but in Q5, a €100 increase in total OOPE was associated with a 0.6% increase in the risk of mortality (Q5: HR 1.006, 95% CI 1.003 to 1.010). CIs overlapped for the coefficients from each income quintile. There were positive associations between total OOPE and mortality in Austria, Spain, Italy and Belgium, with the largest association in Belgium (HR 1.008, 95% CI 1.003 to 1.013); in other countries, no significant association was found.

**Figure 4:**
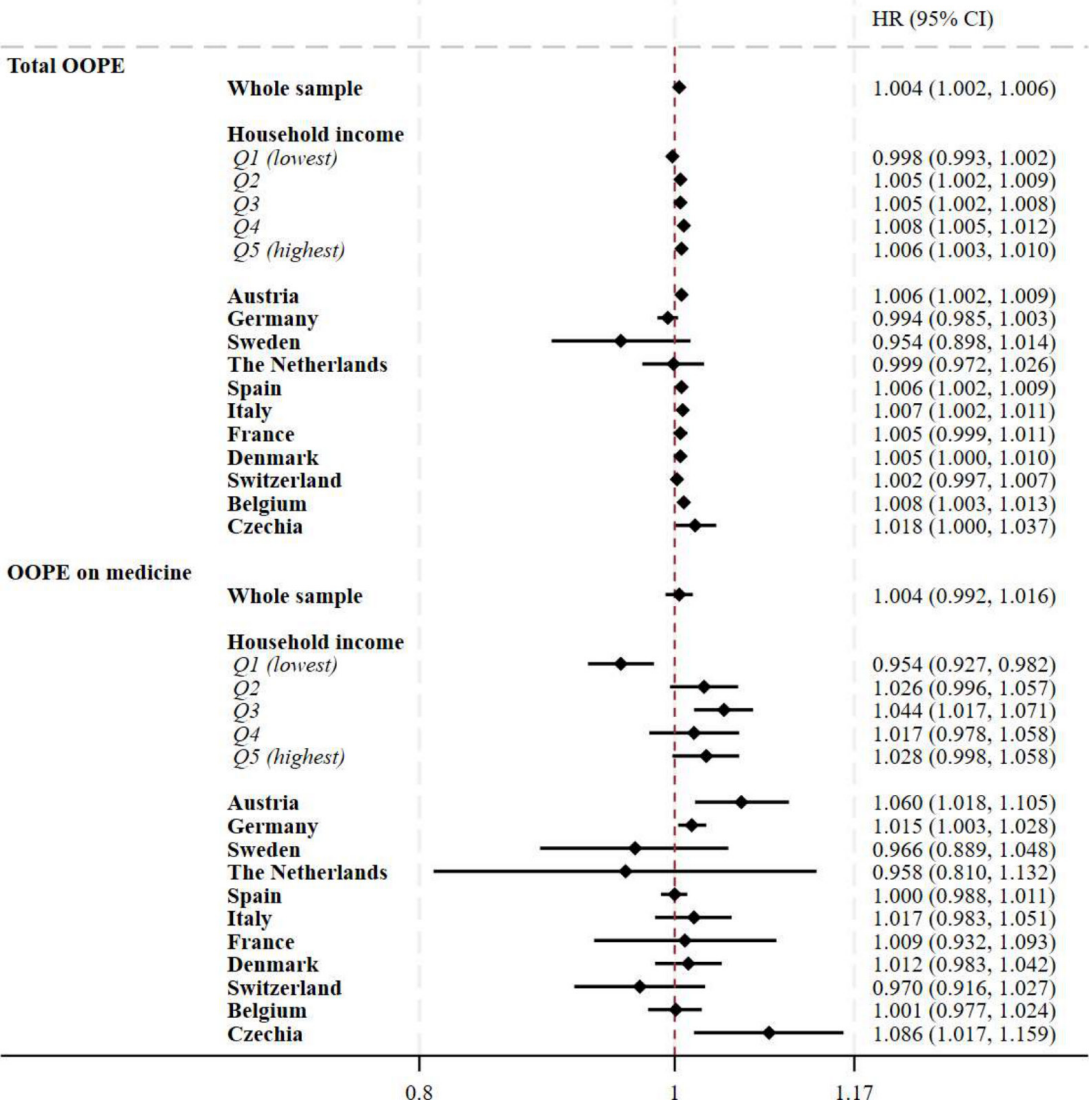
Association between OOPE and mortality in 11 European countries. Notes: the association between OOPE and mortality was modelled using time-varying parametric survival models with Gompertz distribution. Covariates included in the main models were: age (50–59, 60–69 or 70 and older), sex (male, female), BMI (continuous), number of chronic diseases (continuous), marital status (married or in a civil partnership, others), residential country, educational attainment (less than upper secondary, upper secondary or tertiary education), household income (in quintiles within each country for each wave respectively; the poorest being Q1, the richest being Q5), number of primary care visits and hospitalisations in the previous year. Each point estimate and relative CIs in the graph were derived from a separate regression model. HR coefficients were expressed as €100 increase in OOPE. Results in bold had a p value <0.05. BMI, body mass index; OOPE, out-of-pocket expenditure.

In pooled analyses of all countries, higher OOPE on medicines was not associated with an increase in mortality risk. However, this null association was mostly explained by the results stratified by income quintiles, as for those in the lowest quintile a €100 increase in OOPE on medicine was associated with a 15% reduction in mortality risk (95% CI 0.927 to 0.982), while for those in the third quintile the €100 increase in OOPE on medicine was associated with a 4.4% increase in the risk of mortality (95% CI 1.017 to 1.071). Austria, Germany and Czechia were the three countries that reported significant and positive association, with the largest effect found in Czechia (HR 1.086, 95% CI 1.017 to 1.159); in all other countries, no association was reported. Full results are displayed in figure 4.

### Sensitivity analysis

Overall, the IPW-RA model confirmed the results from the main analysis on the association between CHE and mortality over the study period, although the shown effect was generally larger but with wider CIs (online supplemental table 3). A positive association between CHE and mortality was found in Austria, Belgium, Czechia, Denmark, Spain, Switzerland and the Netherlands.

## Discussion

Between 2007 and 2017, on average, 1.6% of the older populations in 11 European countries incurred CHE. The mean annual OOPE per person was approximately €303, with medicines accounting for 27% of this amount (€81). CHE was associated with an increased risk of mortality—a pattern identified across most of the study countries (the greater risk was found in Switzerland). An increase in OOPE exhibited similar but weaker positive associations, with higher total OOPE associated with an elevated risk of mortality. Overall, this relationship demonstrated no substantial variation across income quintiles or between countries (CIs all overlapped). Results were only partially confirmed when focusing on OOPE on medicines, with a negative association found in the poorest quintile (Q1).

The prevalence of CHE in the older populations in 11 European countries identified in this study is lower than other evidence showing the EU average was 4% in 2019.^6^ This may be due to the inclusion of countries with lower average rates of CHE in the study (eg, Netherlands with a CHE equal to 0.5% in 2015; Sweden 1.6% in 2019; Denmark 2.6% in 2015; Spain 2.9% in 2020),^6^ exclusion of countries with high CHE (eg, Portugal 10% in 2015)^44^ or that the study sample is older with a mean age of 65 years. Despite having higher healthcare needs, older populations may be protected from excessive OOPE with accrued wealth and financial resources, or greater coverage of healthcare costs under social protection schemes in Europe. Evidence from Germany and Belgium shows that households with only adults aged 60 year or more had the lowest rates of CHE compared with multigenerational households and households with no one under 60 years of age.^45^

Only 27% of OOPE was due to medicines in our study, a lower estimation than similar studies. Data from 40 countries in the WHO EURO region showed outpatient medicines accounted for 38% of total OOPE.^6^ Again, the study countries may explain these differences, as for countries with lower CHE, total OOPE is driven more by dental care and medical products,^6^ and countries in the EU may directly reduce older households’ exposure to OOPE.

The finding that CHE and total OOPE are associated with increased mortality is plausible given what is known about the health impacts of CHE. CHE and high OOPE are associated with impoverishment,^14^ delay in seeking care,^46^ poor quality of life^15^ and mental health.^16^ There are multiple likely explanations for the association between CHE and mortality. Individuals who have high OOPE and incur CHE may be unhealthier, have costly chronic conditions or have undiagnosed health conditions^810^,^12^—all of which likely increase the risk of death. However, this analysis adjusted for multiple chronic conditions, healthcare use and socioeconomic status (which are associated with underlying health status and propensity to seek healthcare) removed a sizeable proportion of this potential explanatory pathway. Furthermore, if there were biases from unobserved factors, we would expect different associations between CHE and mortality across income quintiles, as lower-income groups typically experience higher rates of poor health, which would contribute to greater exposure to CHE^6^ and higher associated mortality risks. We found no difference in the positive association between CHE and mortality across income quintiles. This may be partially explained by social protection policies in the selected European countries that might mitigate the effect of CHE in more vulnerable groups. This interpretation is supported by previous research, which found that healthcare utilisation in Europe is primarily driven by underlying health needs rather than individuals’ ability to pay. As a result, OOPE may still have a limited effect on access to care, regardless of income level.^47^ Additionally, a higher socioeconomic position is generally associated with access to higher-quality healthcare services, which are often more costly.^48^ Furthermore, IPW-RA sensitivity analyses which also adjusted for the likelihood of incurring CHE (conditional on socioeconomic status, chronic conditions and healthcare use) showed comparable findings, suggesting variation in who incurred CHE may not be a strong explanation. Nonetheless, these analyses could only use the questions available in the surveys, and residual confounding could explain some of the associations found, for example, the timing and reasons for accessing healthcare service that required payment, or whether respondents were benefitting from a social protection scheme that might partially exempt them from payment.

We argue the relationship between CHE and increased mortality is therefore likely driven by the negative health impacts of incurring CHE, including the negative health effects from falling into poverty and reductions in life quality^14 15^ and subsequent delays to seeking care, purchasing necessary medicines and interventions or engaging in health-improving behaviours.^46^ An additional consideration is that personal healthcare costs may be low in older individuals who forgo healthcare due to lack of affordability, complicating the relationship between OOPE and health outcomes.^13 49 50^ Thus, our analysis may be underestimating the true negative impacts of cost-sharing policies for healthcare.

The relationship between OOPE on medicines and mortality across income groups is complex. In our study, higher OOPE for medicines was associated with reduced mortality risk in the lowest income quintile, whereas CHE did not exhibit this pattern. This might suggest that while excessive OOPE negatively impacts health, modest OOPE on medicines in low-income groups may reflect the purchase of essential, life-saving medications. This phenomenon may arise from a prioritisation of critical medicines due to financial constraints, potentially indicating an efficiency in expenditure.^51^ This is supported by a recent European study showing that, under tighter budget constraints, people with multimorbidity reduced spending on other healthcare services more than on medicines.^52^ Conversely, in higher-income groups, low OOPE might signal unmet or undiagnosed medical needs, contributing to increased mortality risk. Costs related to medicine purchasing and adherence to treatments, unlike inpatient or outpatient costs, may be more discretionary, potentially explaining why this pattern was not observed for total OOPE. However, as no specific question in SHARE was asked investigating delayed/forgone care or reasons for purchasing medications requiring OOPE, this assumption cannot be tested in the present study and requires further investigation.

To our knowledge, this is the first study that looked at the association between CHE and mortality, using representative longitudinal data from 11 European countries between 2006 and 2020. There are multiple limitations pertinent to this study. First, the data come from surveys where there may be recall, respondent or response bias, especially among older adults and those from lower socioeconomic groups, who are more prone to underreport information.^10 11^ However, this is likely to have weakened rather than inflated the strength of the association between mortality with expenditures, as previous evidence suggested that individuals with missing mortality data tend to have a higher burden of frailty at baseline, potentially leading to an underestimation of the true association between mortality and financial burden.^53^ Second, although this study goes beyond existing studies on the topic by adjusting for a wide range of socioeconomic and health factors and testing IPW-RA approaches (testing selection bias in likelihood of incurring CHE), there still may be unmeasured confounding which could explain the increased risk of mortality and CHE. For example, unobserved factors such as the timing and reasons for healthcare payments, informal care availability, social support networks and health literacy could influence both the likelihood of incurring CHE and mortality risk. These factors may bias the association upward if individuals with greater health needs both spend more and have higher mortality risk. Third, some country-specific differences may stem from variations in cultural, geographical, political, economic and health system factors, particularly cost-sharing policies, and although we aimed to reduce this residual confounder by including country level fixed effects, this effect may persist—specifically explaining the relationship between expenditures and mortality. For instance, in Switzerland—where we observed particularly higher risk for those who incurred CHE—higher baseline OOPE and reliance on private insurance may amplify the financial burden of illness, leading to greater unmet needs or delayed care. Such structural differences may interact with individual vulnerabilities in ways that fixed effects cannot fully capture. Fourth, we outline multiple potential pathways to explain the relationship between expenditure and mortality (eg, falling into poverty, forgone care, unmet needs, cost-sharing, etc), and disentangling these is beyond the scope of this. Furthermore, there may be interacting and contradicting effects from different pathways, which also likely vary over income groups and countries.

There are key policy implications from this work. The findings emphasise that OOPE remains a significant burden in Europe, with 8% of respondents incurring at least €1000 annually. The associated increases in mortality from increases in OOPE, which were consistent across counties and income groups, paint a worrying picture of newly introduced cost-sharing policies and a broader erosion of UHC.^54^,^56^ While some small OOPE is often used to rationalise demand in universal health systems, tackling the sizeable level of OOPE and removing impoverishing cost-sharing is a necessity. Further research should evaluate the long-term impacts of cost-sharing policies on healthcare utilisation, outcomes and patient satisfaction. Regular monitoring of OOPE, CHE, unmet healthcare needs and forgone care is crucial to mitigate adverse health impacts in European health systems.^54^

## Conclusion

This study highlights the detrimental relationship between high healthcare costs and mortality in older populations across Europe. CHE and high OOPE increased mortality risks, likely through negative impacts on household well-being and forgone care. Policies expanding cost-sharing may disproportionately affect vulnerable groups like older individuals, emphasising the need for continuous monitoring and policy adjustments to ensure equitable access to healthcare.

## Supplementary material

10.1136/bmjph-2025-003228 online supplemental table 1

## Data Availability

Data are available in a public, open access repository.
