# Supplementary material for: Association between catastrophic health expenditure and mortality in older people in 11 European health systems: a longitudinal analysis between 2006 and 2020
Source: BMJ Public Health. 2025 Nov 13;3(2):e003228. doi: 10.1136/bmjph-2025-003228 (PMC12625963; doi:10.1136/bmjph-2025-003228)
Supplement: online supplemental table 1 [file bmjph-3-2-s001.pdf]

Supplemental Material

Supplemental Table 1. Descriptive statistics of the study population at baseline

Notes: all individuals whose information was recorded in at least two waves starting from wave 2 (2006/07) were included in the study. For each participant, the year of the first available interview was considered as baseline year. Cross-sectional survey weights were applied. SD = standard deviation.

	Austria	Belgium	Czechia	Denmark	France	Germany	Italy	Netherlands	Spain	Sweden	Switzerland	Overall
**N**	5091	8165	7623	5344	6539	7365	7354	5273	7893	5911	3809	70367
**Mean follow-up (years)**	3.6 (2.9)	4.0 (3.5)	3.5 (3.1)	4.7 (3.6)	3.8 (3.6)	3.1 (3.1)	4.2 (3.7)	1.9 (2.8)	4.2 (3.7)	4.5 (3.6)	4.3 (3.4)	3.8 (3.4)
**Age (mean (SD))**	65.9 (14.0)	64.4 (14.1)	63.9 (19.3)	63.9 (11.5)	64.2 (13.9)	65.8 (13.8)	65.1 (15.4)	63.9 (18.4)	65.2 (20.0)	65.5 (14.4)	65.1 (12.5)	65.0 (19.8)
**Gender (female)**	54.4%	53.0%	54.5%	52.0%	54.0%	54.1%	53.9%	51.8%	53.7%	51.7%	52.6%	53.7%
**Marital Status**												
Married/Partnership	61.2%	68.4%	63.4%	65.7%	65.1%	65.1%	69.9%	67.6%	71.9%	63.1%	67.7%	67.0%
Single	9.0%	6.4%	3.2%	7.6%	9.2%	6.9%	8.4%	8.3%	8.3%	9.0%	7.1%	7.9%
Divorced/Widowed	29.9%	25.2%	33.4%	26.8%	25.7%	28.1%	21.8%	24.2%	19.8%	28.0%	25.2%	25.2%
**Education Attainment**												
No formal education	0.1%	2.0%	0.1%	0.1%	11.9%	1.0%	7.5%	0.8%	15.2%	0.6%	0.2%	6.2%
Primary school	15.3%	18.0%	12.4%	13.3%	22.3%	0.7%	37.3%	13.1%	39.2%	24.6%	11.1%	20.4%
Secondary and higher	84.6%	79.9%	87.4%	86.6%	65.7%	98.2%	55.3%	86.0%	45.6%	74.8%	88.8%	73.4%
**Income**												
Q1 (poorest)	21.7%	19.9%	21.5%	18.5%	21.4%	20.6%	22.3%	19.5%	21.8%	19.4%	20.8%	21.2%
Q2	19.3%	18.5%	19.0%	18.3%	19.4%	19.4%	18.2%	19.2%	17.9%	19.1%	18.9%	18.9%
Q3	19.5%	19.0%	19.4%	19.4%	18.8%	19.4%	19.0%	19.3%	19.6%	19.9%	19.8%	19.2%
Q4	19.5%	21.1%	19.5%	21.1%	19.6%	20.0%	19.4%	20.3%	19.0%	20.4%	20.0%	19.7%
Q5 (richest)	20.0%	21.5%	20.6%	22.7%	20.8%	20.6%	21.1%	21.7%	21.7%	21.2%	20.6%	21.0%
**Job status**												
Retired	60.0%	42.8%	60.2%	43.0%	48.6%	50.2%	45.0%	35.2%	35.1%	50.0%	41.0%	46.3%
Employed/Self-employed	24.2%	33.0%	32.3%	44.5%	35.5%	31.9%	28.3%	37.0%	26.6%	43.6%	43.8%	32.1%
Unemployed	2.4%	4.2%	3.2%	3.1%	3.8%	4.7%	3.2%	2.0%	6.5%	1.9%	1.7%	4.0%
Permanently sick/disabled	1.2%	5.6%	3.0%	5.8%	3.4%	3.0%	2.5%	7.4%	4.5%	2.4%	2.7%	3.4%
Homemaker/Other	12.2%	14.4%	1.3%	3.6%	8.7%	10.2%	21.0%	18.4%	27.3%	2.1%	10.9%	14.2%
**BMI (mean (SD))**	26.9 (6.3)	26.4 (5.4)	27.7 (9.2)	25.9 (4.6)	26.1 (5.3)	26.9 (5.7)	26.1 (5.2)	26.1 (6.0)	27.3 (8.1)	26.0 (5.1)	25.6 (4.8)	26.5 (7.7)
**Number of CDs (mean (SD))**	1.4 (1.8)	1.7 (1.9)	1.8 (2.7)	1.6 (1.6)	1.6 (1.7)	1.7 (1.9)	1.7 (2.1)	1.2 (1.8)	1.6 (2.6)	1.5 (1.7)	1.1 (1.4)	1.6 (2.6)
**Number of primary care visits in previous year (mean (SD))**	8.5 (15.8)	8.1 (12.2)	7.8 (13.9)	4.7 (7.8)	6.7 (9.8)	8.6 (14.4)	8.4 (14.2)	5.2 (11.4)	7.2 (17.1)	3.5 (7.4)	5 (8.8)	7.5 (17.9)
**Number of hospitalisations in previous year (mean (SD))**	0.4 (1.3)	0.3 (1)	0.3 (1.5)	0.2 (0.7)	0.3 (1)	0.3 (0.9)	0.2 (0.9)	0.2 (0.7)	0.2 (1.7)	0.2 (1)	0.3 (1.1)	0.3 (1.3)

Supplemental Table 2. Out-of-pocket expenditure at baseline

Notes: out-of-pocket expenditure was calculated as the amount spent in the year before the baseline interview for each participant. Survey weights were applied. OOPE = out-of-pocket expenditure.

	Mean OOPE (€)			No OOPE			Lower than 100 euros			Between 100 and 999 euros			Between 1,000 and 9,999 euros			equal or higher than 10,000 euros		
	Mean	(95%CI)		Proportion	(95%CI)		Proportion	(95%CI)		Proportion	(95%CI)		Proportion	(95%CI)		Proportion	(95%CI)	
**COUNTRY**																		
**Overall**	303.4	291.2	315.6	39.6%	39.2%	40.0%	19.2%	18.9%	19.6%	33.5%	33.1%	33.9%	7.5%	7.3%	7.7%	0.2%	0.1%	0.2%
**Household income**																		
Q1	247.2	225.3	269.2	37.2%	35.8%	38.6%	22.7%	21.3%	24.0%	34.4%	33.0%	35.9%	5.6%	4.9%	6.3%	0.1%	0.0%	0.3%
Q2	307.5	271.5	343.4	30.2%	28.8%	31.6%	24.0%	22.6%	25.4%	38.7%	37.2%	40.2%	6.9%	6.0%	7.7%	0.2%	0.0%	0.4%
Q3	290.8	261.5	320.2	28.6%	27.3%	30.0%	25.5%	24.1%	26.9%	39.5%	38.0%	40.9%	6.2%	5.5%	7.0%	0.2%	0.0%	0.3%
Q4	310.2	277.6	342.9	27.3%	26.1%	28.5%	26.2%	24.7%	27.7%	39.5%	38.1%	40.8%	6.9%	6.1%	7.6%	0.2%	0.0%	0.4%
Q5	361.5	328.9	394.1	27.5%	26.2%	28.7%	24.6%	23.2%	25.9%	39.1%	37.4%	40.7%	8.7%	7.9%	9.5%	0.2%	0.0%	0.5%
**Austria**	421.6	383.1	460.1	26.0%	24.5%	27.5%	20.4%	18.9%	21.9%	44.4%	42.7%	46.2%	8.9%	8.1%	9.8%	0.3%	0.2%	0.6%
**Belgium**	515.7	493.6	537.8	7.5%	6.7%	8.3%	22.6%	21.4%	23.8%	55.1%	53.7%	56.4%	14.8%	13.9%	15.8%	0.1%	0.0%	0.1%
**Czechia**	91.2	85.8	96.6	15.7%	14.3%	17.2%	56.8%	54.9%	58.7%	27.3%	25.7%	28.9%	0.3%	0.2%	0.4%	0.0%		
**Denmark**	328.0	311.3	344.7	14.8%	13.8%	15.9%	22.1%	20.9%	23.3%	55.7%	54.3%	57.1%	7.5%	6.8%	8.3%	0.0%		
**France**	187.5	164.3	210.7	46.4%	45.0%	47.9%	27.1%	25.9%	28.4%	22.2%	21.1%	23.4%	4.1%	3.6%	4.7%	0.1%	0.0%	0.2%
**Germany**	286.0	258.0	314.1	16.4%	15.4%	17.5%	31.9%	30.6%	33.2%	46.5%	45.1%	47.8%	5.0%	4.5%	5.6%	0.2%	0.1%	0.5%
**Italy**	453.4	421.0	485.9	26.5%	25.2%	27.8%	14.4%	13.4%	15.5%	47.0%	45.5%	48.4%	11.8%	11.0%	12.8%	0.3%	0.2%	0.7%
**Netherlands**	150.2	134.7	165.7	52.8%	50.7%	54.8%	18.5%	17.0%	20.2%	24.8%	23.2%	26.6%	3.9%	2.9%	5.2%	0.0%		
**Spain**	218.3	183.5	253.1	50.9%	49.0%	52.9%	21.9%	20.4%	23.5%	22.3%	20.7%	24.0%	4.7%	3.9%	5.8%	0.2%	0.1%	0.5%
**Sweden**	349.1	334.0	364.1	7.8%	6.8%	8.8%	14.6%	13.5%	15.8%	72.3%	70.8%	73.7%	5.4%	4.8%	6.1%	0.0%		
**Switzerland**	691.6	635.2	748.0	22.5%	21.0%	24.1%	10.3%	9.3%	11.5%	47.7%	45.9%	49.4%	19.4%	18.1%	20.7%	0.2%	0.1%	0.4%

Supplemental Table 3. Association between catastrophic health expenditure and mortality in 11 European countries between 2006 and 2020.

Notes: The association between catastrophic health expenditure and mortality was modelled using time-varying parametric survival models with Gompertz distribution with inverse probability weighting regression adjustment. Covariates included in the main models were: age (50–59, 60–69, or 70 and older), sex (male, female), BMI (continuous), number of chronic disease (continuous), marital status (married or in a civil partnership, others), residential country, educational attainment (less than upper secondary, upper secondary, or tertiary education), household income (in quintiles within each country for each wave respectively; the poorest being Q1, the richest being Q5), number of primary care visits and hospitalisations in the previous year. Propensity score of encountering CHE was calculated employing logit models adjusted for age, gender, marital status, education attainment, household income, number of chronic diseases, number of primary care visits and hospitalisations in the previous year, and country of residence. In bold results that had a p-value <0.05. HR = hazard ratios; 95%CI = 95% confidence intervals.

Mortality	HR	95%CI	
Whole sample	4.07	2.32	7.16
Stratified analysis			
Q1	7.34	3.05	17.63
Q2	2.64	1.60	4.35
Q3	2.01	1.17	3.45
Q4	10.85	6.39	18.43
Q5	1.29	0.41	4.10
Austria	7.14	2.84	17.98
Belgium	9.03	5.50	14.84
Czechia	5.58	3.35	9.30
Denmark	2.25	1.30	3.90
France	1.14	0.70	1.86
Germany	2.21	0.88	5.59
Italy	2.04	0.80	5.19
Spain	4.93	2.34	10.41
Sweden	1.46	0.75	2.84
Switzerland	6.39	2.05	19.93
The Netherlands	6.75	2.60	17.55
